# Membranes for Bone Engineering Enriched with Magnesium- and Strontium-Substituted Hydroxyapatite

**DOI:** 10.1021/acsomega.6c01548

**Published:** 2026-07-16

**Authors:** Marco Antônio Rigo Rodrigues, Harley Oliveira Guedes, Myllene Bossolani Galloro, Ana Carolina Cabral de Medeiros, Giovanne Delechiave, Giovanna Sarra, Larissa Tavares Sampaio Silva, Juliana Kelmy Macário Barboza Daguano, Luiz Henrique Catalani, Luiz Henrique da Silva Nali, Maria Stella Moreira, Letícia Cidreira Boaro, Flávia Gonçalves

**Affiliations:** † 154625 Universidade Ibirapuera, Departamento de Odontologia, Av. Interlagos 1329 − 4° Andar, São Paulo, São Paulo 04661-100, Brazil; ‡ Universidade Santo Amaro, Pós-Graduação em Odontologia, R. Prof. Enéas de Siqueira Neto, 340, São Paulo, São Paulo 04829-300, Brazil; § 28133 Instituto de Química da Universidade de São Paulo, Departamento de Química Fundamental, Av. Prof. Lineu Prestes, 748, São Paulo, São Paulo 05508-000, Brazil; ∥ Faculdade de Odontologia da Universidade de Sao Paulo, Av. Prof. Lineu Prestes 2222, São Paulo, São Paulo 05508-000, Brazil; ⊥ Centro de Engenharia, Modelagem e Ciências Sociais Aplicadas (CECS), Universidade Federal do ABC, Av. dos Estados, 5001, Santo André, São Paulo 09210-580, Brazil; # Departamento de Estomatologia, AC Camargo Cancer Center, São Paulo, São Paulo 01509-010, Brazil; ∇ University of Saskatchewan, College of Dentistry, 105 Wiggins Rd, Saskatoon, Saskatchewan S7N5E5, Canada; ○ Universidade Santo Amaro, Pós-Graduação em Ciências da Saúde, R. Prof. Enéas de Siqueira Neto, 340, São Paulo, São Paulo 04829-300, Brazil

## Abstract

Hydroxyapatite doped with magnesium and strontium can be a promising strategy for improving bone regeneration. The aims of this study were (1) to synthesize hydroxyapatite (HA) with magnesium (Mg^2+^) and/or strontium (Sr^2+^) substitution and to characterize them; (2) to develop electrospun scaffolds associating HA synthesized with poly-l-lactide (PLLA); and (3) to evaluate the osteoinductive and osteoconductive potential of these scaffolds with ionic changes, associated with human periodontal ligament stem cells (hPDLSC). HA was synthesized with ionic substitution with Mg^2+^, Sr^2+^, or both ions and confirmed by X-ray diffraction analysis. Scanning electron microscopy (SEM) showed HA crystals with nanometric size. Membranes of PLLA and PLLA_HA with conventional or ionic substitution were obtained by electrospinning and evaluated by proliferation (CCK-8), differentiation (alizarin red) and qPCR assays with hPDLSC in osteogenic and clonogenic media. Data were subjected to two-way and one-way ANOVA with Tukey’s test, and Kruskal–Wallis with Student–Newman–Keuls test (α=0.05). The ionic substitutions of HA did not influence adhesion and proliferation at 3 or 7 days. However, in clonogenic medium scaffold containing ionic substitutions presented greater extracellular mineralization than the control PLLA. Furthermore, scaffolds with HA and magnesium presented higher expression of osteopontin. In the osteogenic medium, only the material with HA conventional or with both ions changes presented greater OPN expression than the control material and greater extracellular matrix mineralization. It can be concluded that membranes containing HA_Mg_Sr have higher osteogenic properties even in clonogenic conditions, improving hPDLSC differentiation and becoming a promising alternative for bone regeneration applications.

## Graphical abstract

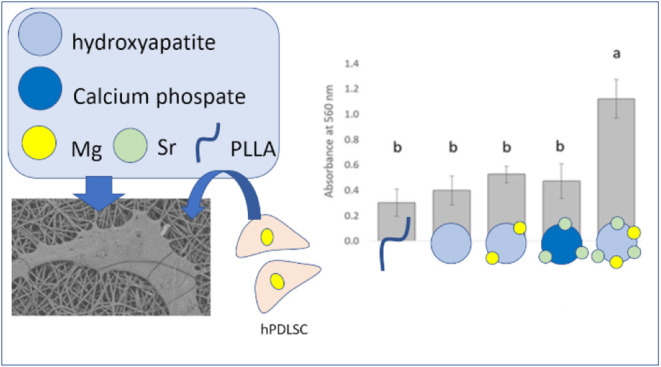

## 1 Introduction

The association of a scaffold, stem cells, and bioactive molecules is needed for bone regeneration in tissue engineering. The scaffolds can be developed in several forms, dimensions, and compositions, but they should present mechanical properties and degradation rate compatible with the target tissue.^1^ In general, they are composed of osteoconductive natural or synthetic polymers, which barely present inductive or osteogenic properties.^2^ However, inorganic content such as bioactive glasses and hydroxyapatite can be added to form composite biomaterials with some osteoinductive potential.^3^

Owing to its presence in the bone mineral phase, hydroxyapatite (HA) has been the material of choice for bone repair for many years in medical and dental applications.^4^ In addition to its promising biological properties, the slow degradation rate of HA is a limiting factor for its use in pure form.^5^ Its use associated with natural and synthetic polymers, however, has been effective for bone regeneration in both in vitro and in vivo studies,^6,7^ given that they combine the advantages of both materials.

The chemical and biological properties of HA can be improved by the replacement of calcium with magnesium or strontium ions.^8,9^ The incorporation of magnesium has shown direct and indirect effects on mineralized tissues.^10^ Directly, it favors osteoblast activity and proliferation, improving osteointegration, whereas its deficiency promotes a low degree of inflammation and increased osteoclast activity, contributing to bone loss.^11^ Indirectly, magnesium affects parathyroid hormone secretion and, consequently, the secretion of 1,25-dihydroxyvitamin D.^12^

A significant amount of strontium is also observed in calcified tissues and in newly formed bone in vivo.^13^ Low doses of strontium contribute to adequate bone formation, whereas high doses can be associated with osteomalacia.^13^ The administration of strontium ranelate induces osteoblast differentiation and decreases bone resorption, inhibiting osteoclast activity.^14^

In this way, the synthesis of polymeric scaffolds associated with magnesium- and strontium-substituted HA would be a promising alternative to bone engineering when associated with stem cells. Therefore, the objectives of this study were (1) to synthesize HA using the conventional formulation and magnesium and strontium substitution; (2) to develop and characterize polymeric membranes of poly-l-lactide (PLLA) associated with these synthesized hydroxyapatites; and (3) to associate the developed membranes with mesenchymal stem cells (MSCs) and to evaluate proliferation, osteoinduction, and osteodifferentiation properties in vitro. The study hypothesis is that partial replacement of calcium with magnesium and strontium ions in hydroxyapatite associated with PLLA scaffolds decreases proliferation and increases extracellular matrix mineralization and expression of bone metabolism-related genes due to greater osteoconduction and osteodifferentiation of stem cells.

## 2 Materials and Methods

### 2.1 Synthesis and Characterization of Hydroxyapatite

Four syntheses were performed to produce hydroxyapatite (Table 1): conventional HA, ionic substitution with magnesium, ionic substitution with strontium, or magnesium- and strontium- cosubstitution. For hydroxyapatite synthesis, 0.03 M diammonium hydrogen phosphate (Sigma-Aldrich, St. Louis, USA) solution (pH 11) was dropped in 0.05 M calcium nitrate (Sigma-Aldrich) solution (pH 11) and kept under agitation at 90 °C for 5 h in a nitrogen atmosphere. For ionic substitution, part of the calcium nitrate solution was replaced with magnesium nitrate or strontium nitrate (Sigma-Aldrich). The final solution was sonicated for 10 min, and the precipitate was collected by centrifugation at 5,000 rpm for 5 min, washed three times with distilled water, and dried in an oven at 37 °C for 24 h.

**Table 1:** Molar Concentrations Used in the Syntheses of Conventional Hydroxyapatite (HA), Magnesium-Substituted HA (HA_Mg), Strontium-Containing Calcium Phosphates (CP_Sr), or Magnesium- and Strontium-*Co*-Substituted HA (HA_Mg_Sr)

	Solution 1 (50% vol)	Solution 2 (50% vol)		
Material	Diammonium hydrogen phosphate (M)	Calcium nitrate (M)	Strontium nitrate (M)	Magnesium nitrate (M)
HA	0.03	0.05	-	-
HA_Mg	0.03	0.0425	-	0.0075
CP_Sr	0.03	0.0425	0.0075	-
HA_Mg_Sr	0.03	0.0425	0.00375	0.00375

All synthesized material samples were analyzed by X-ray powder diffraction (Miniflex Model, Rigaku, Austin, USA) with CuKα radiation, λ=1.54183 Å, and Ni filter. The diffractograms were obtained with 2θ scans from 0° to 90°, in increments of 0.01° and scan speed of 0.1 deg/min. The data were analyzed by Crystallographic Search-Match (International Union of Crystallography Chester, England) to identify the phases present and were then plotted using OriginPro 8.5 (OriginLab Corporation, Massachusetts, USA).

The magnesium and strontium ionic concentrations in the samples were determined by inductively coupled plasma optical emission spectrometry (ICP-OES). The intensities observed were compared to a calibration curve for the concentration of each chemical element evaluated.

### 2.2 Synthesis and Characterization of Membranes

The polymeric membranes associated with calcium phosphate-synthesized materials were made by electrospinning. A polymeric solution was obtained by solubilizing 5 wt % PLLA (Purasorb PL 32, Corbion, Holland) in chloroform. Calcium phosphates were added to the solution at 10 wt % in relation to the PLLA mass and sonicated for 15 min in 0.5 mL of chloroform. Finally, dimethylformamide was added to the final solution (10 vol %) and electrospun according to the following parameters: 3 mL/h flow rate, 18 cm distance between needle and collector, and 20 kV. Five membranes were synthesized, a control of pure PLLA and four membranes by associating the PLLA to the four calcium phosphate formulations presented in Table 1.

The morphology of calcium phosphates and of electrospun mats was analyzed by scanning electron microscopy (SEM, FEG 7401F, Jeol, Tokyo, Japan) before and after cell adhesion. The samples were previously dried in a vacuum desiccator and sputtered with gold to a thickness of 6 nm. The diameter of the fibers was measured using ImageJ software (National Institutes of Health, Maryland, USA) using at least 50 measurements for each material.

### 2.3 Isolation and Characterization of hPDLSC

This study was approved by the Research Ethics Committee of Ibirapuera University (protocol CAAE no. 04312818.0.0000.5597). Primary cultures of human periodontal ligament stem cells (hPDLSCs) were obtained by the explant technique from fragments of periodontal ligaments of human permanent teeth.^15^ All of the experiments were limited to five passages, and all cell culture procedures were done in a laminar flow cabinet, following the sterility protocols for materials and solutions (NBR/IEC 601.2.22 and IEC 60825–1/2001–8 safety standards).

The isolated MSCs were characterized by flow cytometry to verify whether they presented stem cell characteristics. The following primary antibody panel was used: conjugated with fluorescein (FITC), phycoerythrin (PE), or allophycocyanin (APC) against human surface molecules, namely, CD90-APC, CD105-FIT (BD Biosciences, CA, USA), CD146-APC (Biolegend, CA, USA), STRO-1-FITC, CD44-FITC, CD 1-FITC, and the not uncoupled antibodies from the manufacturers—CD31-FIT, CD14-PE, and CD45-APC—and their isotype controls IgG1k-FITC and IgG2k-FITC (all from BD Biosciences, CA, USA).

The cell cultures were expanded to a minimum of 1 × 10^6^ cells per surface marker. The experiment was performed at 4 °C, and the centrifugations were performed at 550x g for 5 min. The culture plates were maintained in a cold container. The cells were washed twice in PBSA and collected using a proteolytic marine enzyme (StemPro Accutase; GIBCO) for 10 min. The cells were centrifuged, suspended, and fixed in 10 mL of 4% paraformaldehyde. The cell suspension was marked with a primary antibody and, whenever necessary, incubated with a secondary antibody for fluorophore coupling. The samples were counted and classified in the flow cytometer (FACSCalibur, Becton Dickinson, CA, USA). At least 50,000 events were acquired within the gate, and the data were analyzed by FlowJo Software version 9.6.2 (Tree Star, OR, USA).

### 2.4 Cell Proliferation Assay

Cell Counting Kit-8 (CCK-8, Sigma-Aldrich) assay was used to evaluate the effect of the membranes on the cell viability and proliferation at 3 and 7 days. hPDLSCs in passage 3 were placed on the top of the membranes with density of 2 × 10^4^ cells/well. After 3 and 7 days of culture, cell viability was evaluated using the Cell Counting Kit-8 (CCK-8, Sigma-Aldrich). The membranes were removed from the wells and transferred onto a new plate, preventing interference of nonadherent cells. A solution of 450 μL of culture medium and 50 μL of CCK-8 solution was added to each well and incubated for 3 h. Supernatants (50 μL) were collected, and their absorbance was read at 450 nm on a spectrophotometer (ELx-800, Biotek, Winooski, USA). Two scaffolds from each group after 3 days were processed and observed by SEM for analysis of cell morphology and adhesion. Cells cultured on the wells served as the positive control, whereas scaffolds with no cells served as the negative control.

### 2.5 Osteodifferentiation Assays

Stem cell differentiation was evaluated by two assays: quantitative polymerase chain reaction (qPCR) and by the alizarin red assay, both in cultures with osteogenic and clonogenic medium.

For qPCR, the following genes related to bone metabolism were evaluated: runt-related transcription factor 2 (RUNX2), osteopontin (OPN), and osteocalcin (OCN) in cultures at 7 and 21 days.

RNA was extracted using the DNeasy Kit (Qiagen GmbH, Hilden, Germany), and the samples were treated with DNase enzyme using the DNase I – Amplification grade (Life Technologies Corporation, Carlsbad, USA), following the manufacturer’s instructions. The reverse transcriptase enzyme was used to synthesize cDNA from mRNA using the SuperScript III First-Strand Synthesis System (Life Technologies Corporation). qPCR was performed using Power SYBR Green PCR Master Mix (Thermo Fisher, Vilnius, Lithuania) for 40 cycles at 95 °C for 15 s and 60 °C for 1 min in a 96-well format on a Step One Plus real-time PCR system (Applied Biosystems). Relative quantification was determined based on the mean cycle threshold (CT) values of triplicate samples. GAPDH was used as an internal reference gene to normalize the expression levels of the target genes. Relative expression was calculated in comparison to the expression of PLLA samples at 7 or 21 days of culture, and the results were expressed in fold change by the 2-ΔΔct method. The primers used are listed in Table 2.

**Table 2:** Primers Sequence for the Reverse and Forward Directions

Gene	Direction	Primers sequence
GAPDH	F	5′ AAGGTGAAGGTCGGAGTCAAC 3′
	R	5′ GGGGTCATTGATGGCAACAATA 3′
RUNX2	F	5′ CCTTTACTTACACCCCGCCA 3′
	R	5′ GGTCCTGACGAAGTGCCAT 3′
OPN	F	5′ GTGATGTCCTCGTCTGTAGCATCA 3′
	R	5′ GTAGACACATATGATGGCCGAGG 3′
OCN	F	5′ ATTGTGGCTCACCCTCCATC 3′
	R	5′ CCAGCCTCCAGCACTGTTTA 3′

For the alizarin red assay, hPDLSCs were added over the membranes at a density of 1 × 10^5^ cells/well. The cells were cultured for 21 days in two different culture media – a clonogenic medium (CM) or an osteogenic differentiation medium (ODM). The ODM was composed of high-glucose Dulbecco’s Modified Eagle’s medium (Embryolife, Campinas, Brazil), supplemented with 10% SFB, 1% penicillin/erythromycin (10,000 U mLmL^–1^), 10 mM β-glycerol phosphate (Sigma-Aldrich), 50 μg/mL ascorbic acid (Sigma-Aldrich) and 10^–9^ M dexamethasone (Sigma-Aldrich). After 21 days, the membranes were washed twice with PBS and fixed in 10% paraformaldehyde for 10 min. The staining solution was prepared by solubilizing 1% alizarin red in a 2% ethanol solution.

The membranes were immersed in the staining solution for 3 min, washed abundantly in deionized water, and desorbed in 10% cetylpyridinium chloride (Sigma-Aldrich) for 30 min. The supernatant was collected, and absorbance was measured at 560 nm on a spectrophotometer. The membranes were also cultured without cells in both culture media to serve as a control of the initial calcium content present in the membrane samples.

### 2.6 Statistical Analysis

Following normality and homoscedasticity requirements, the proliferation and alizarin red data were subjected, respectively, to two-way and one-way ANOVA and Tukey’s test. Data from qPCR were submitted to the Kruskal–Wallis test, and means were compared using Student–Newman–Keuls test. In all the analyses, it was considered a 95% significance level (α=0.05).

## 3 Results

XRD analysis confirmed that HA is the main phase in the synthesis of these compounds (JCPDS PDF no. 09–432), except for CP_Sr (Figure 1A). The HA sample presented high crystallinity, characterized by narrow and intense diffraction peaks. When substitutions of Ca^2+^ with Mg^2+^ and Sr^2+^ occurred, the base peaks were larger because of distortions generated in the net parameters. These distortions occurred for the diffraction planes, such as (002) at 26.0°, (211) at 31.8°, (112) at 32.2°, (300) at 32.9°, and (310) at 39.9°. The distortions were greater in CP_Sr samples, causing the disappearance of some peaks and appearance of others, leading to the formation of new crystalline phases (Figure 1B). Figure 1C shows the diffractogram for CP_Sr with the identification of all crystalline phases. In this case, the HA phase was not observed, except for the ones resulting from Ca^2+^ substitution with Sr^2+^, namely, strontium calcium hydroxyphosphate (Ca5Sr5­(PO4)­6­(OH)­2 - JCPDS PDF no. 34–479), strontium hydrogen phosphate (SrHPO_4_-JCPDS PDF no. 33–1335), and strontium hydroxyapatite (Sr5­(PO4)­3­(OH)–JCPDS PDF no. 33–1348). A diffraction peak at 36.8° suggests the presence of zircon, an indicative of material contamination.

**Figure 1:**
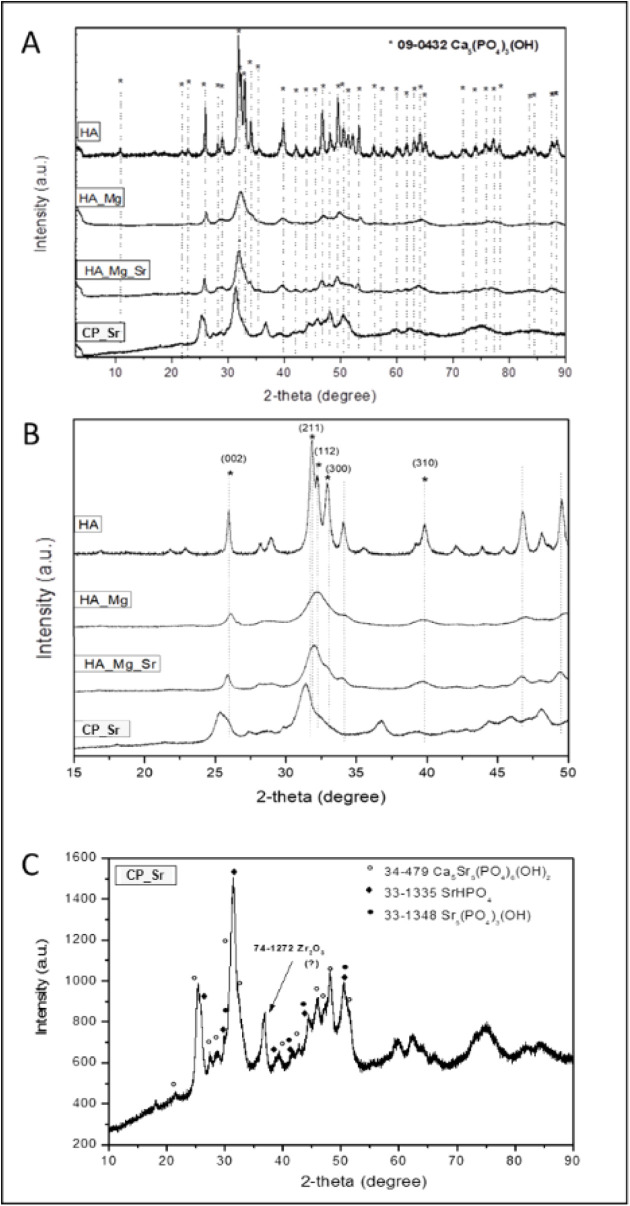
X-ray diffraction pattern. (A) General view of HA, HA_Mg, HA_Mg_Sr, and CP_Sr. (B) Detailed diffraction planes (002), (211), (112), (300), and (310) for HA, HA_Mg, HA_Mg_Sr, and CP_Sr. (C) CP_Sr sample with all crystalline phases.

According to the ICP-OES analysis, the rates of magnesium and strontium incorporated into HA or CP crystals were, respectively, 3.2% and 12.1% when added individually or 1.5% and 4.3% when incorporated concomitantly.

The morphology of HA and CP with or without ionic modifications was similar—needle-shaped crystals with a nanometric size, as observed by SEM (Figure 2A to 2D). HA crystals were aggregated under the form of corals.

**Figure 2:**
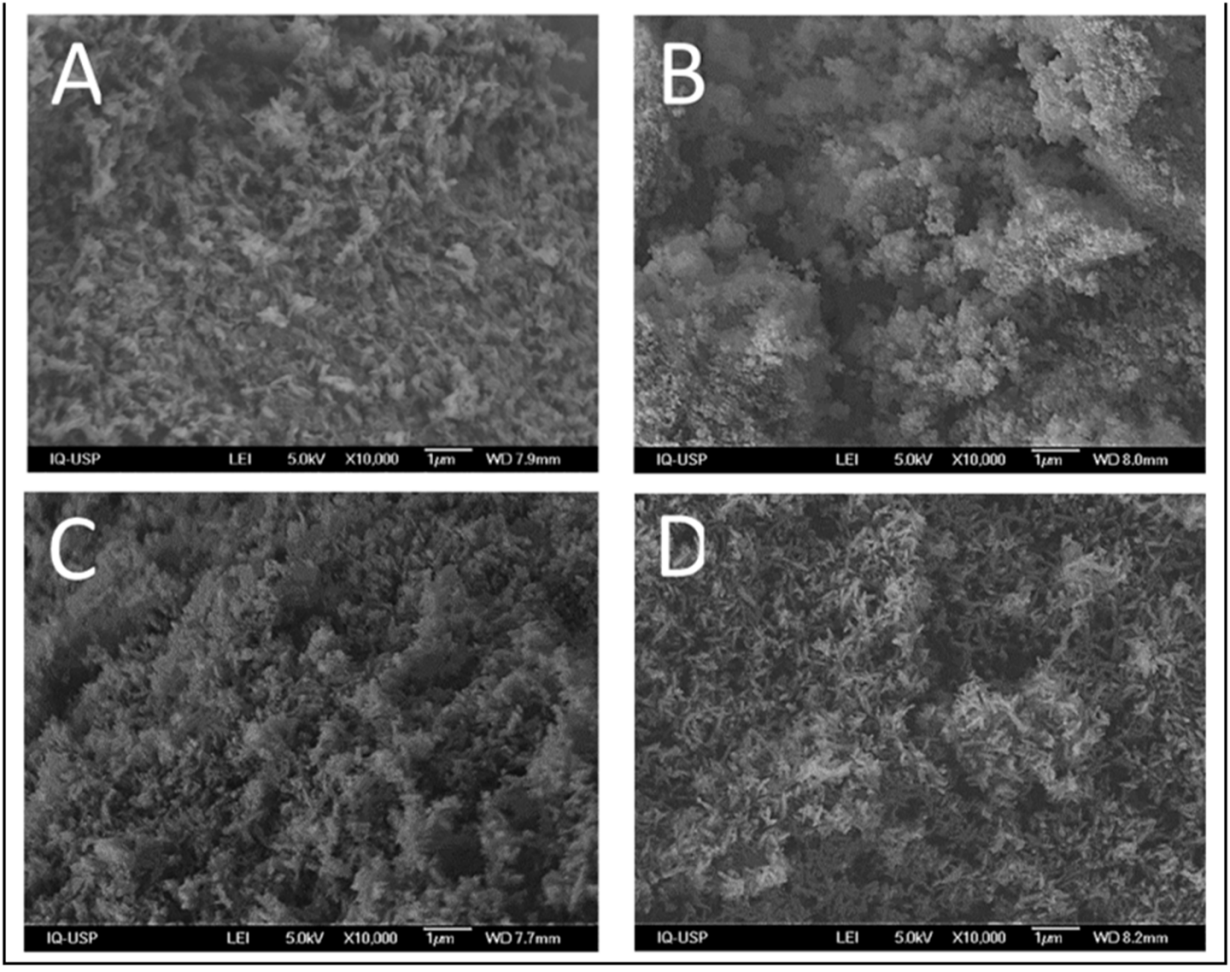
Microscopy of synthesized samples: (A) hydroxyapatite; (B) magnesium-substituted hydroxyapatite; (C) strontium-containing calcium phosphates; (D) magnesium- and strontium-*co*-substituted hydroxyapatite.

The electrospun mats of PLLA and the synthesized calcium phosphates showed fibers with a mean diameter of 2 μm, randomly scattered in a three-dimensional net (Figure 3). There was no significant difference in morphology and deposition of fibers for the different calcium phosphates added.

**Figure 3:**
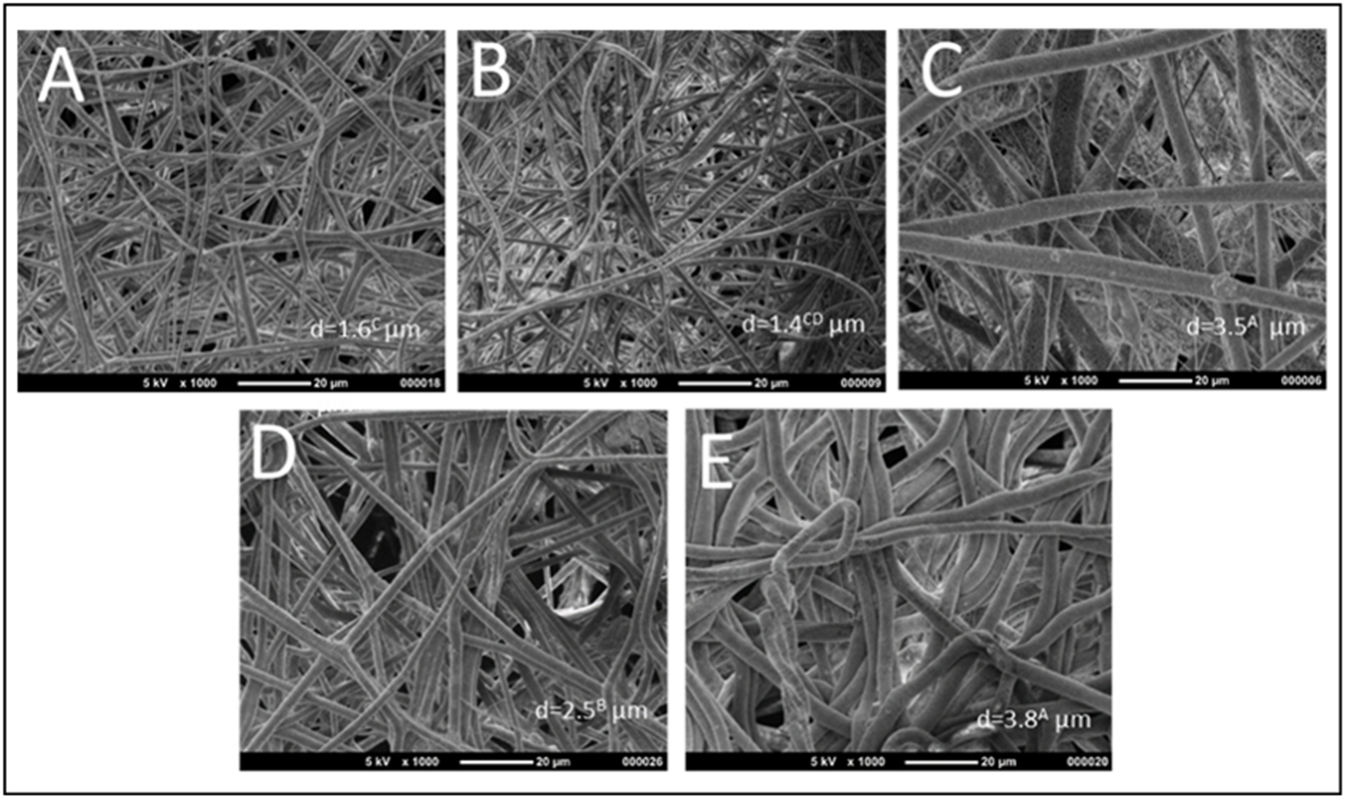
SEM (x1000) of electrospun mats. (A) PLLA; (B) PLLA and hydroxyapatite; (C) PLLA and magnesium-substituted hydroxyapatite; (D) PLLA and strontium-modified calcium phosphates; (E) PLLA and magnesium- and strontium-*co*-substituted hydroxyapatite.

Cell characterization of the first lineages of hPDLSCs revealed a typical mesenchymal stem cell (MSC) immunophenotype. Cell markers for mesenchymal and undifferentiated cells were positively expressed (CD146, CD90, STRO-1, CD44, and CD105), while hematopoietic and endothelial cells (CD14, CD31, and CD45) were minimally or not expressed at all.

The rates of positive cells were 60.8% for CD146, 0.67% for STRO-1, 96.3% for CD44, 98.9% for CD105, and 99.8% for CD90, confirming the mesenchymal profile of the hPDLSC population. In contrast, low expression levels were observed for hematopoietic and endothelial markers (2.88% for CD14, 1.13% for CD31, and 3.67% for CD45).

At 3 and 7 days, no statistically significant differences in cell proliferation were observed among the different materials (*p* > 0.05). However, a significant increase in cell proliferation over time was detected in all groups, from 3 to 7 days (Figure 4). In all experimental groups, the scaffold surfaces supported cell adhesion and spreading, with cells exhibiting a flattened morphology and filopodia extensions anchored to the fibers as early as day 3 (Figure 5).

**Figure 4:**
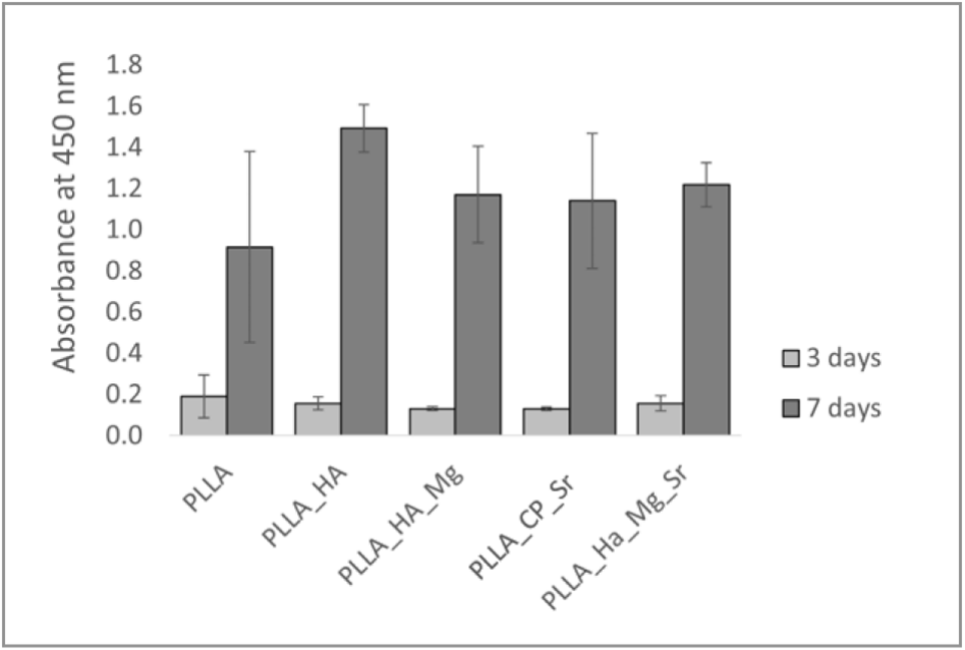
Mean and standard deviation of absorbance at 450 nm relative to CCK-8 proliferation assay at 3 and 7 days. At 3 and 7days: *p* > 0.05 and no difference among groups (two-way ANOVA and Tukey test, α = 0.05).

**Figure 5:**
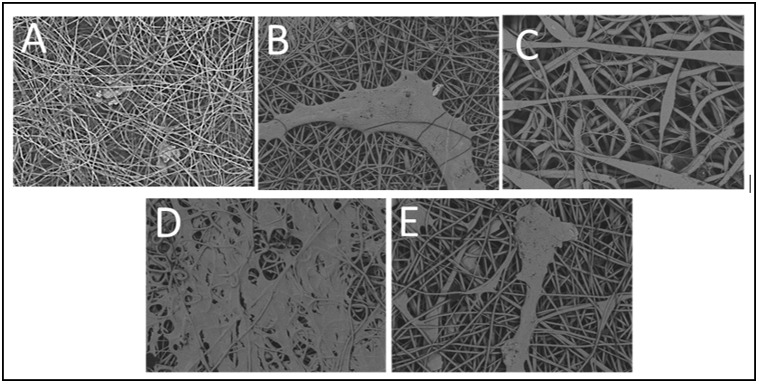
Scanning electron microscopy (SEM, × 1000) of electrospun mats showing cell adhesion and spreading of hPDLSCs on scaffold surfaces. (A) PLLA; (B) PLLA and hydroxyapatite; (C) PLLA and magnesium-substituted hydroxyapatite; (D) PLLA and strontium-modified calcium phosphates; (E) PLLA and magnesium- and strontium-*co*-substituted hydroxyapatite.

When hPDLSCs were cultured in osteogenic medium, the expression levels of RUNX2 and Osteocalcin did not change among the experimental materials and the PLLA control at 7 days (Figure 6a and Figure 7A). Likewise, no differences in RUNX2 expression were observed among the experimental materials at 21 days compared to PLLA (Figure 6B). The expression of OPN was greater than the control in materials PLLA_HA conventional or PLLA_HA_Mg_Sr (Figure 8B) at 21 days. In the clonogenic medium, PLLA_HA_Mg presents lower RUNX2 expression than the control in 7 days (Figure 6C), and PLLA_CP_Sr or PLLA_HA_Mg_Sr showed lower expression than PLLA_HA at 21 days (Figure 6D). Regarding OCN, the material PLLA_HA_Mg showed lower expression than PLLA_HA and PLLA_CP_Sr at 7 days (Figure 7C) but higher expression than PLLA_HA and PLLA_HA_Mg_Sr in 21 days at clonogenic medium (Figure 7D). Also, the PLLA_HA_Mg presented greater expression of OPN than the control material at 21 days (Figure 8D). OPN expression of PLLA_HA_Mg_Sr was statistically similar to all the other materials at 7 and 21 days in clonogenic medium.

**Figure 6:**
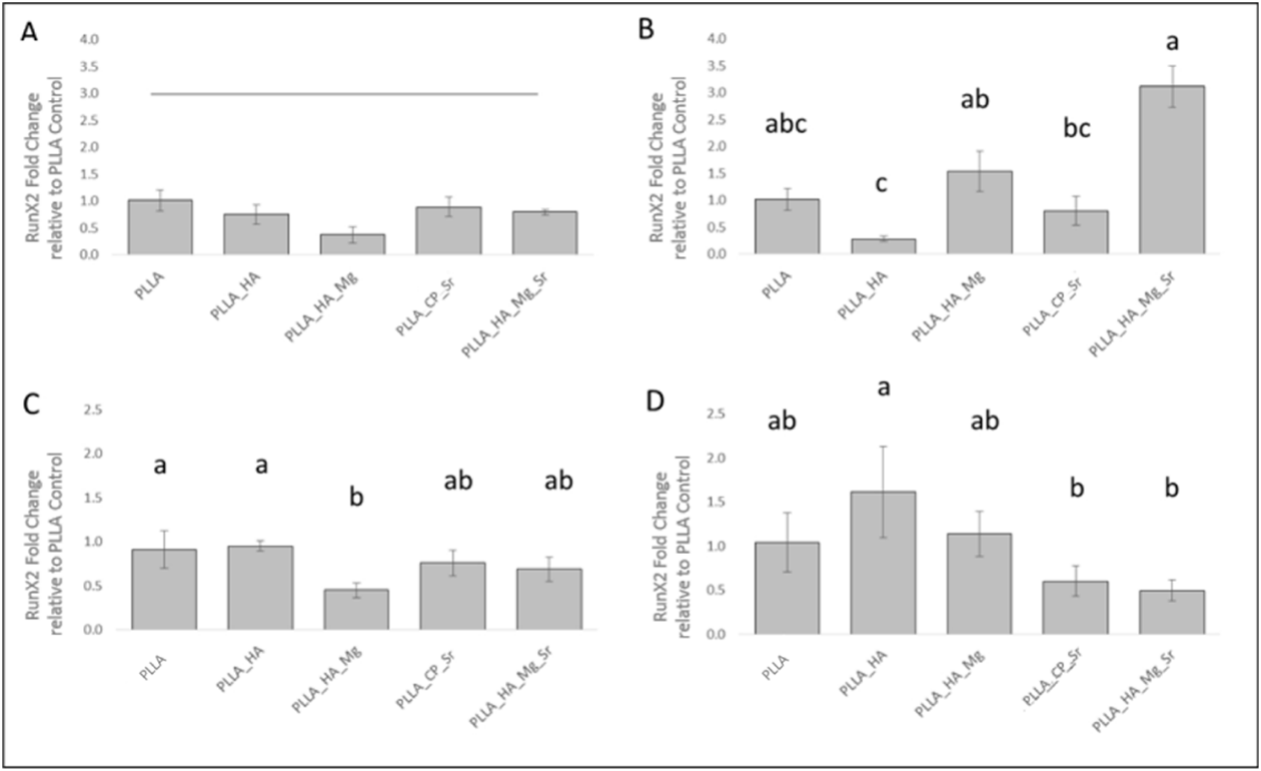
Mean and standard deviation of relative expression of the RUNX2 gene in relation of hPDLSC cultured in PLLA membranes using osteogenic medium (A and B) or clonogenic medium (C and D) at 7 (A and C) and 21 days (B and D). GAPDH was used as an internal control. Similar letters indicate no significant difference. Horizontal lines over the bars indicate the absence of statistical differences among all the groups (Kruskal–Wallis and Student–Newman–Keuls test, α = 0.05).

**Figure 7:**
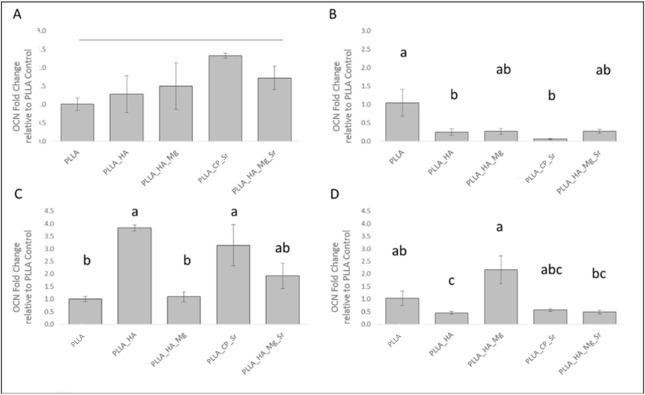
Mean and standard deviation of relative expression of the osteocalcin (OCN) gene in hPDLSC cultured in PLLA membranes using osteogenic medium (A and B) or clonogenic medium (C and D) at 7 (A and C) and 21 days (B and D). GAPDH was used as an internal control. Similar letters indicate no significant difference. Horizontal lines over the bars indicate the absence of statistical differences among all the groups (Kruskal–Wallis and Student–Newman–Keuls test, α = 0.05).

**Figure 8:**
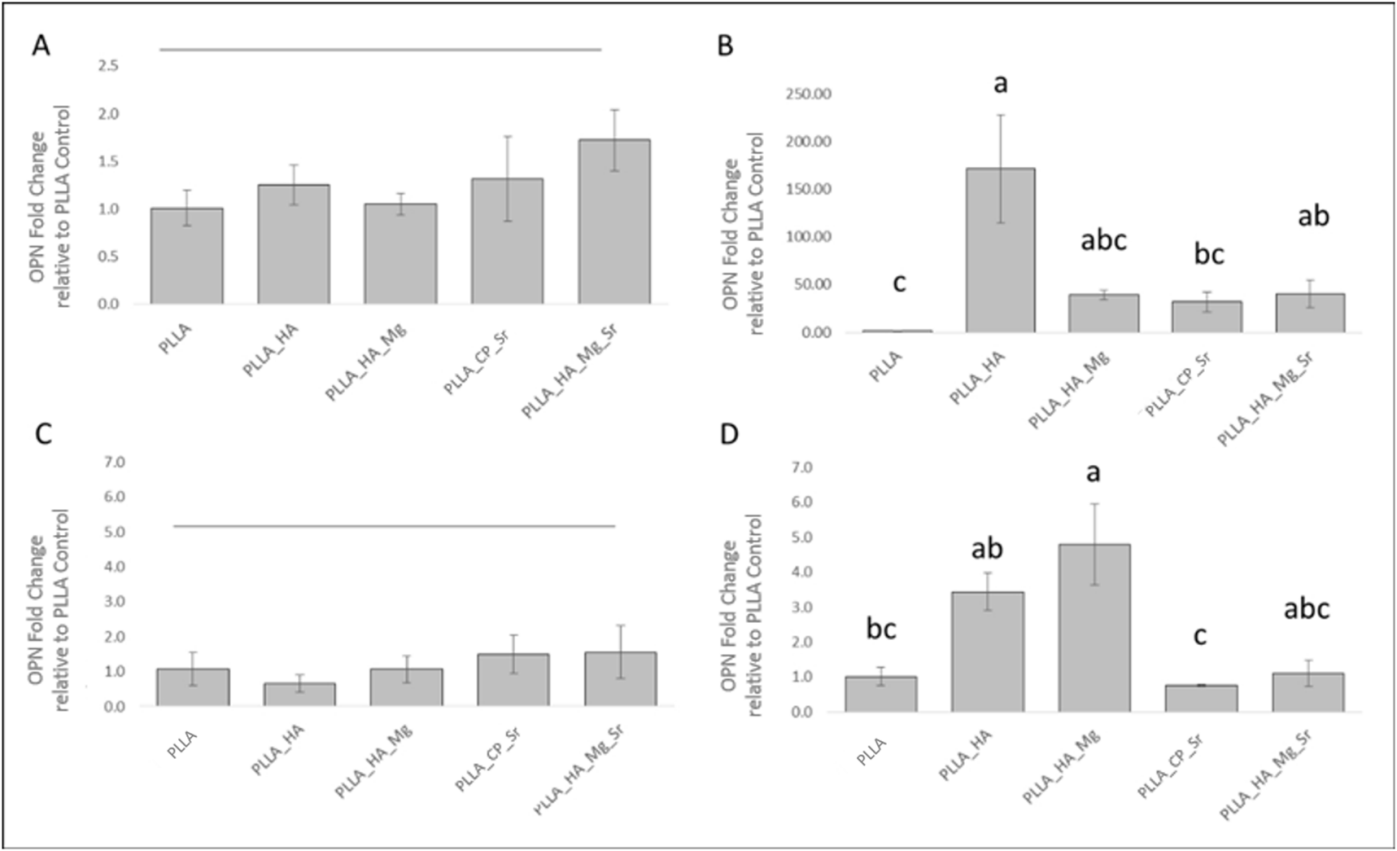
Mean and standard deviation of relative expression of the osteopontin (OPN) gene in hPDLSC cultured in PLLA membranes using osteogenic medium (A and B) or clonogenic medium (C and D) at 7 (A and C) and 21 days (B and D). GAPDH was used as an internal control. Similar letters indicate no significant difference. Horizontal lines over the bars indicate the absence of statistical differences among all the groups (Kruskal–Wallis and Student–Newman–Keuls test, α = 0.05).

In the osteogenic differentiation medium, the scaffold with greater mineralization of the extracellular matrix was PLLA_HA_Mg_Sr. All the other scaffolds were statistically similar to the PLLA control scaffold (Figure 9A). In the clonogenic medium, scaffolds of PLLA_HA_Mg, PLLA_CP_Sr, and PLLA_HA_Mg_Sr presented statistically greater mineralization of the extracellular matrix than the PLLA control group, whereas PLLA_HA scaffolds showed mineralization of the extracellular matrix similar to all the other groups (Figure 9B).

**Figure 9:**
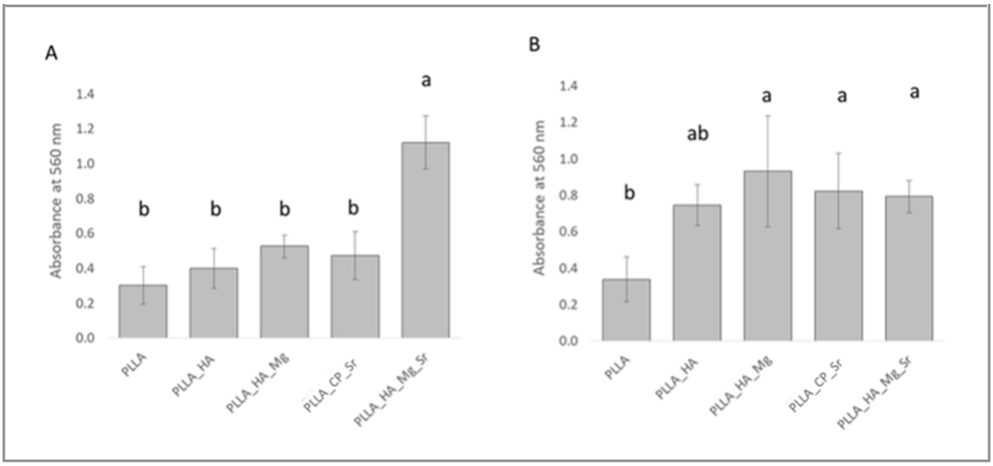
Mean and standard deviation of absorbance at 560 nm relative to the alizarin red assay bounded to calcium in scaffolds with (A) hPDLSCs cultured in osteogenic medium for 21 days; and (B) hPDLSCs cultured in clonogenic medium for 21 days. Similar letters indicate no significant difference (One-way ANOVA and Tukey test, α=0.05).

## 4 Discussion

In this study, the synthesis of nanometric HA or calcium phosphate phases with ionic modification with magnesium and strontium ions enabled the effective development of electrospun polymeric membranes of PLLA associated with several kinds of these calcium phosphates. The cultured hPDLSC presented greater osteogenic differentiation in the membranes containing ionic-substituted HA, when cultured in the osteogenic medium and compared to the PLLA control group, showing a promising biomaterial for use in regenerative dentistry.

The crystalline phases present in all synthesized materials were determined by XRD analysis, and the diffractograms were in accordance with the previous protocols described in the literature for HA,^16^ except for CP_Sr, which does not correspond to a single-phase strontium-substituted hydroxyapatite but rather to a multiphasic system composed of Ca_5_Sr_5_(PO_4_)_6_(OH)_2_, SrHPO_4_, and Sr_5_(PO_4_)_3_(OH). Therefore, its biological behavior should be interpreted considering the combined effects of these distinct crystalline phases. Although the presence of Sr-rich phases may enhance ionic release and bioavailability of Sr^2+^ ions, and differences in solubility among these phases may result in a more dynamic ion release profile compared to stoichiometric hydroxyapatite, potentially influencing cell–material interactions over time, this material presented an intermediary performance and does not stand out in any of the biological tests performed. In addition, the presence of zirconium oxide in the CP_Sr sample is attributed to contamination from the zirconia mortar and pestle used during the powder processing step after drying. Zirconium oxide is widely reported as a biocompatible material with no cytotoxic effects.^17^ Furthermore, previous studies have demonstrated its potential to enhance osteoblast adhesion, proliferation, and differentiation,^18^ as well as to modulate the expression of genes related to bone metabolism.^17,19^ Despite these findings, the PLLA_CP_Sr material did not exhibit superior performance compared to the other groups in any of the conducted assays. This suggests that the observed contamination did not confer any measurable functional advantage and should therefore be considered a limitation of the study.

Regarding the hydroxyapatite phases in the synthesized materials, it can be observed that the ionic substitutions caused small variations in diffractogram planes, which can also be associated with the low crystallinity of the precipitated material, as magnesium can inhibit HA nucleation.^16^ The amount of substituted calcium was different for each synthesis. The lower incorporation of magnesium compared to that of strontium was expected and can be attributed to atomic size, crystalline structure, oxidation state of the atoms,^8^ and the inhibitory effect on HA crystallization.^16,20^ Although both cations present the same valence, the effective ionic ray of the magnesium ion (0.69 Å) is lower than that of calcium (0.99 Å), as previously reported in the literature;^21^ consequently, it becomes closer to or within the limit of adjustment of the apatite grade.^21^ On the other hand, strontium incorporation was higher. This ion showed great affinity for bone HA and can substitute 100% of the calcium ion without changing its structure,^22^ generating other composites. Furthermore, its chemical similarity to calcium allows it to act as a calcium receptor agonist, activating several intracellular signaling pathways that contribute to bone remodeling.^22^ In addition, based on the present data, the incorporation of Mg^2+^ appears to play a critical role in modulating the phase composition, favoring a more homogeneous substitution within the apatite structure and reducing the formation of secondary Sr-rich phases when cosubstitution with strontium is performed.

The use of MSCs from the human periodontal ligament has shown good potential for proliferation and bone differentiation in vitro and in vivo studies.^23^ The immunophenotypic characterization of the isolated cells exhibited a similar profile to that of hPDLSC, as previously described in the literature.^24^

Materials biocompatibility is a key factor in cell therapy and tissue engineering, and it is directly related to the biological behavior of the cells when in contact with the scaffold. Adhesion is the first interaction to occur, followed by the natural spread of the cells on the material. Therefore, initial cell adhesion will influence not only the proliferation ability but also differentiation.^25^ The fixation of cells on the scaffolds increases survival time and cell viability.^26^ Besides the ionic difference, all PLLA scaffolds obtained from electrospinning, generating a mat without beads and with homogeneous fiber distribution; in other words, all of them developed membranes, contributing to cell adhesion and growth. No statistically significant difference was observed among the materials in the initial adhesion and proliferation at 3 and 7 days. Also, all the materials presented larger cell proliferation from 3 to 7 days. The absence of statistically significant differences in cell proliferation among the groups indicates that ionic substitution does not impair early cytocompatibility. Although the initial hypothesis proposed a potential reduction in proliferation, this effect was not observed within the evaluated time frame (3–7 days). This finding may be explained by the fact that mesenchymal stem cells are still in an early stage of differentiation during this period, in which proliferation remains active.^27^ Indeed, previous studies have shown that a decrease in proliferation is typically associated with later stages of osteogenic differentiation and matrix mineralization (around 14–21 days).^28^ Importantly, these results suggest that the effects of ionic substitution are more likely related to the modulation of osteogenic differentiation rather than early proliferative responses, reinforcing that the observed differences in mineralization are driven by bioactive ionic effects rather than variations in initial cell viability.

In the clonogenic medium, the effect of the material on gene expression, compared with the PLLA control group, was more pronounced than that in the osteogenic medium. Notably, the PLLA_HA_Mg material exhibited a lower RUNX2 expression than both the control group and conventional HA at 7 days. However, at 21 days, it showed higher OPN expression than the control group and higher OCN expression than that of conventional HA, suggesting enhanced osteogenic activity at later stages.

RUNX2 is a key transcription factor involved in osteoblastic differentiation,^29^ typically highly expressed during the early stages of osteogenesis and progressively downregulated as cells transition toward a more mature phenotype and mineralization advances.^30,31^ Previous studies have demonstrated that RUNX2 expression peaks during the early differentiation phase (around 3–7 days) and decreases at later stages (14–21 days), concomitant with increased expression of late markers such as OPN and OCN.^28,30,31^

In this context, the reduced RUNX2 expression observed at 7 days in the PLLA_HA_Mg group may reflect two possible scenarios: either a lower level of early osteogenic commitment or a more advanced progression along the differentiation pathway with cells having already passed the peak of RUNX2 expression. Considering the concomitant increase in late-stage markers (OPN and OCN) at 21 days, the latter interpretation is supported by the overall temporal gene-expression profile. Nevertheless, this interpretation should be considered with caution as no protein-level or functional assays were performed to directly confirm the differentiation stage.

Conversely, OPN and OCN are associated with extracellular matrix mineralization and are predominantly expressed in the intermediate and late stages of bone differentiation, respectively.^32,33^ Indeed, studies have shown that magnesium increases the expression of bone markers in mesenchymal stem cells (MSCs) in a dose-dependent manner,^34^ up to a certain limit, ultimately leading to greater bone neoformation.^35^ However, regarding the extracellular matrix mineralization evaluated by the alizarin red assay, the materials containing HA or CP, regardless of the type, were similar to each other, while materials with ionic substitutions promoted greater differentiation than the control material. This suggests that factors beyond gene expression contribute to the extracellular mineralization. In fact, the osteoinductive potential of HA has been demonstrated both in vitro^36,37^ and in vivo,^38^ independent of ionic modifications.

When hPDLSCs were cultured in osteogenic medium, no experimental material stimulated or inhibited the expression of RUNX2, OCN, or OPN compared to the PLLA control group at 7 days, indicating that initial gene expression was primarily driven by the culture medium itself. The material’s effect was observed in the later stages of gene expression (21 days). The material containing hydroxyapatite modified with magnesium and strontium, which exhibited the highest extracellular matrix mineralization in the alizarin red assay, also demonstrated increased OPN expression compared to the control group at 21 days, indicating a greater osteogenic capacity in these materials, in agreement with the literature.^39,40^ Osteopontin functions as a mediator that promotes the binding of cells to the inorganic matrix,^32^ influences mesenchymal stem cell proliferation and migration, and plays a role in bone tissue formation and remodeling.^41^ Although osteocalcin is directly related to extracellular matrix mineralization,^33^ its expression in the PLLA_HA_Mg_Sr material did not differ from the control. This apparent discrepancy suggests that mineral deposition is not solely dependent on osteocalcin transcription levels since post-transcriptional and translational mechanisms can significantly influence protein abundance.^42,43^ In addition, four different forms of osteocalcin are known based on the degree of carboxylation.^44^ Furthermore, osteocalcin is a secreted protein that becomes incorporated into the extracellular matrix, and its contribution to mineralization may not be accurately reflected by gene expression at a single time point.^45^ The temporal dynamics of osteogenic markers further complicate this relationship, as OCN expression may not coincide precisely with peak mineral deposition.^46^ Furthermore, the presence of Mg^2+^ and Sr^2+^ ions may enhance mineralization through mechanisms that are partially independent of OCN expression, including modulation of cellular activity and extracellular matrix mineral deposition,^47–50^ as will be discussed further.

In osteogenic medium, conventional HA did not promote significantly greater mineralization compared to the PLLA control, which may appear unexpected given the previously reported osteogenic potential of hydroxyapatite.^51^ However, the biological performance of HA is highly dependent on its physicochemical characteristics, including crystallinity and particle size,^52,53^ as well as its dispersion within the polymeric fibers. In electrospun mats, HA particles may be partially embedded within the PLLA fibers, limiting their direct interaction with cells and reducing their effective bioactivity. Additionally, the presence of osteogenic supplements in the culture medium may attenuate the detectable differences between groups.

In contrast, the enhanced mineralization observed for the PLLA_HA_Mg_Sr group suggests that dual ion substitution plays a significant role in modulating cellular behavior, creating a synergistic effect that amplifies osteogenic responses beyond what either ion achieves alone or compared to stoichiometric HA. This synergistic mechanism involving complementary cellular signaling pathways (strontium enhances osteogenic differentiation by activating the Wnt/β-catenin signaling pathway,^47^ while magnesium activates the canonical Wnt signaling pathway).^48^ Also, the cosubstitution can optimize ion release kinetics, as it alters crystal nucleation and growth behavior.^49^ Magnesium incorporation reduces crystallinity and creates calcium-deficient hydroxyapatite, which enhances solubility and degradation properties in a dose-dependent manner,^49^ increasing the release of both ions and its potential effects on cell differentiation. Furthermore, strontium is able to increase matrix mineralization in a dose-dependent way, altering the composition and release of matrix vesicles, which affects the initiation of the mineralization process.^50^

Considering that magnesium and strontium have a direct and an indirect effect on osteogenesis^11–13^ , and also in osteoclasts activity,^54–56^ future studies are needed to evaluate the indirect effects of these ionic substitutions and their in vivo effects, since it has shown promising results in bone regeneration.

## 5 Conclusion

Given the limitations of this in vitro study, it can be concluded that all hydroxyapatite- and CP_Sr-based materials were able to promote the osteodifferentiation and osteoinduction of hPDLSCs. Magnesium- or strontium-*co*-substituted HA associated with PLLA was able to improve the mineralization of the extracellular bone matrix and, consequently, the bone differentiation of hPDLSCs in the osteogenic medium.

## Data Availability

The data sets generated during and/or analyzed during the current study are available in the Deposita Dados repository, https://doi.org/10.48472/deposita/VNITUY.
